# Progress in research on the effects of quinoa (*Chenopodium quinoa*) bioactive compounds and products on intestinal flora

**DOI:** 10.3389/fnut.2024.1308384

**Published:** 2024-02-28

**Authors:** Honglan Huang, Chengxuan Jia, Xinying Chen, Li Zhang, Yang Jiang, Xianglong Meng, Xianjun Liu

**Affiliations:** ^1^College of Biological and Food Engineering, Jilin Engineering Normal University, Changchun, China; ^2^Department of Gastrointestinal Colorectal and Anal Surgery, The China-Japan Union Hospital of Jilin University, Changchun, China; ^3^Department of Burns Surgery, The First Hospital of Jilin University, Changchun, China

**Keywords:** intestinal flora, grain product, quinoa, SCFA, polyphenol compounds, saponins, polysaccharides, bioactive peptides

## Abstract

Quinoa is a highly nutritious whole-grain crop with unique values as both a food and medicinal supplement. At present, the roles played by the intestinal microflora in human health are gaining considerable attention from the research community, and studies to date have shown that the occurrence of a range of diseases may be associated with an imbalance of the intestinal flora. The bioactive compounds of quinoa affect the production of SCFAs and the adjustment of intestinal pH. In this article, we review the mechanisms underlying the effects of different quinoa constituents on the intestinal flora, the effects of these constituents on the intestinal flora of different hosts, and progress in research on the therapeutic properties of quinoa constituents, to provide a better understanding of quinoa in terms its dual medicinal and nutritional properties. We hope this review will provide a useful reference for approaches that seek to enhance the composition and activities of the intestinal flora.

## 1 Quinoa

**Figure 1:**
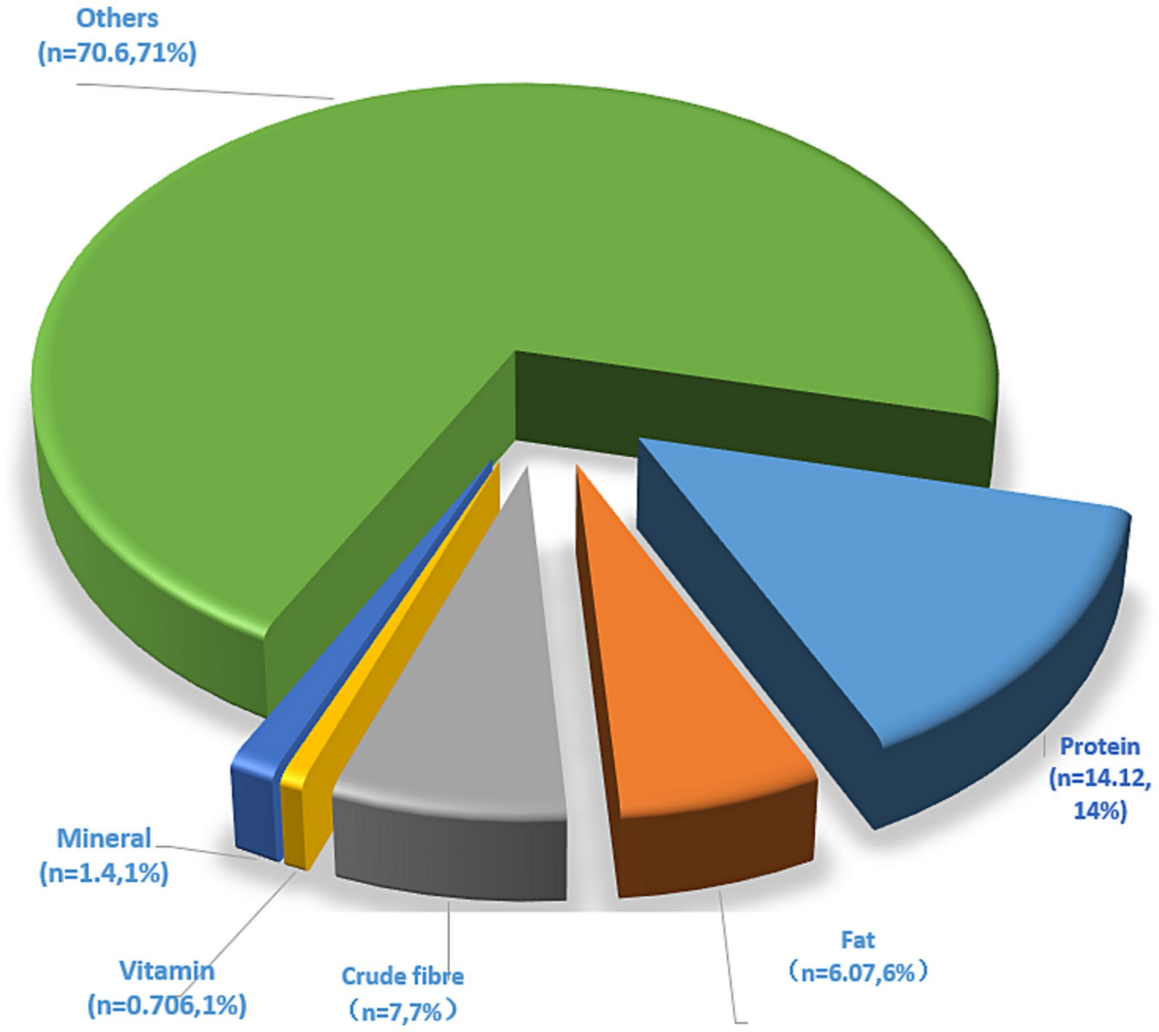
Nutrients of quinoa. Data were obtained from the US Department of Agriculture database. In the figure, *n* represents the proportion of this substance.

**Figure 2:**
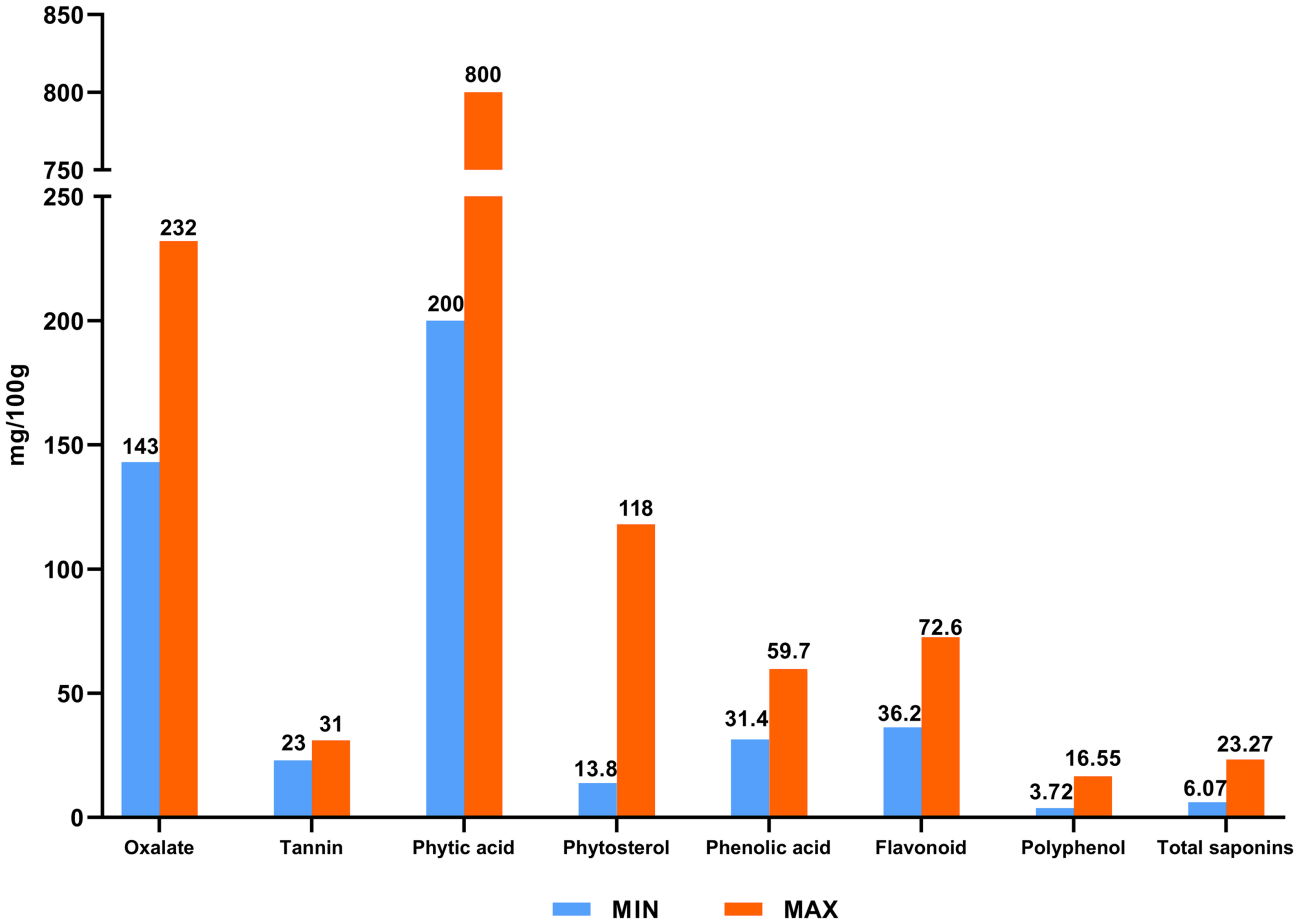
Bioactive substances of quinoa.

*Chenopodium quinoa*, a plant originating from the Andes Mountains of South America, can be divided into white, red, and black color varieties. It is a hardy plant noted for its broad environmental tolerance to low temperatures, drought, and saline–alkaline conditions, and has become a traditional food crop on account of its excellent cold tolerance and ability to grow in high-altitude regions. Today, *C. quinoa* is mainly cultivated in South America and Asia, and in China, it is grown primarily in Shanxi and Qinghai provinces, and the northwest of the country. It is noted as a particularly nutrient-rich grain crop and a valuable source of proteins, fat, minerals, vitamins, fiber, and other nutrients, the levels of which tend to be higher than those in more common cultivated grain crops (1, 2). Quinoa is considered a whole-grain product containing a rich variety of bioactive substances, including phenols, saponins, and glucans, and can play potentially essential roles in the treatment of different diseases (3–5), based on the anti-oxidative, anti-cancer, anti-radiation, antibacterial and cholesterol metabolism-regulatory effects of its constituent (6). Figure 1 shows the nutrient contents of quinoa and Figure 2 presents a profile of the associated bioactive substances, which have been widely described in previous studies (6–13).

**Figure 3:**
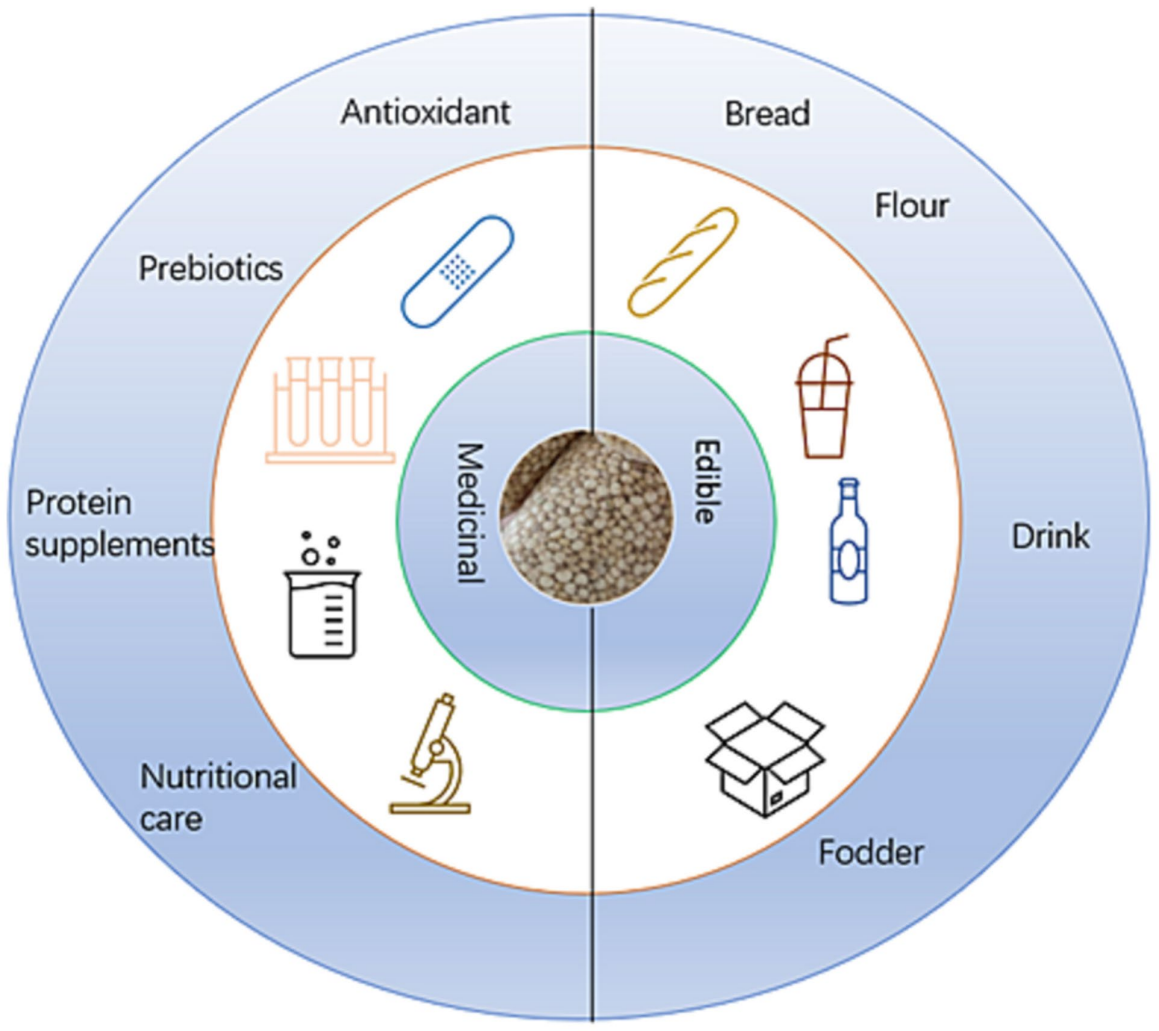
Usage of quinoa (13–15).

Quinoa is considered an example of a plant with dual nutritional and medicinal properties, in that it can either be consumed as a food or applied for therapeutic purposes, depending on the context (Figure 3). In addition to being consumed as a whole grain, quinoa can be processed to produce a diverse range of food products, including yogurt, bread, noodles, biscuits, ice cream, porridge, and meal replacement powders. However, despite its medicinal value, such as in the supplementation of amino acids, there has to date been comparatively little in-depth research to assess the potential medicinal uses of quinoa and its therapeutic effects (13–15).

**Figure 4:**
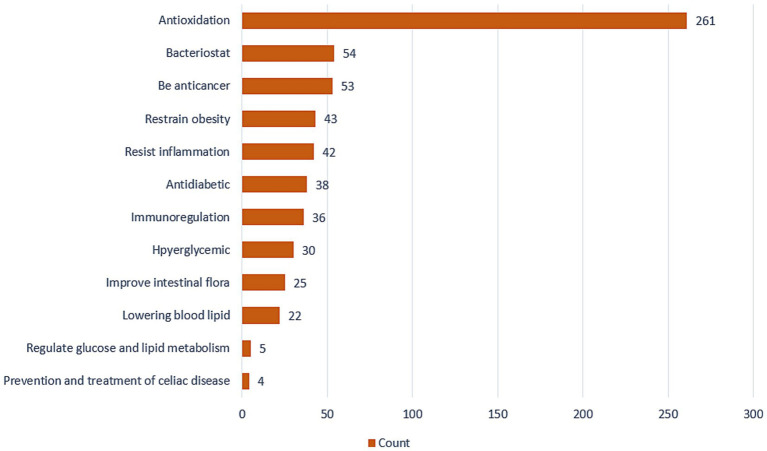
The number of studies on the active functions of quinoa.

In the past few years, there has been an increase in research assessing the application of quinoa in the treatment of liver cancer, obesity, diabetes, colon cancer, and other diseases (Figure 4). In this context, recent discoveries regarding the mechanisms underlying gut–brain–liver andintestinal flora –brain interactions have led to an increasing recognition of the importance of intestinal flora as a mediator in the body’s resistance and response to disease. It is accordingly speculated that the beneficial effects of consuming quinoa may be associated with an enhancement of the health and activities of gut microbiota (16–29).

### 1.1 Intestinal flora

Intestinal flora is a key component of the microenvironments of the small and large intestines. It is a dynamic and complex microbial system that plays essential roles in host metabolism, the occurrence of disease, and immune regulation (30). The principal bacterial taxa comprising intestinal flora include *Bacteroidetes*, *Actinobacteria* (mainly *Bifidobacterium*), *Proteobacteria* (mainly *Enterobacter*), and *Firmicutes* (mainly *Lactobacillus*, *Enterococcus*, and *Clostridium*). The outer mucosal layer of the colon is primarily populated by bacteria in the phyla *Firmicutes*, *Bacteroidetes*, *Actinobacteria*, *Proteobacteria*, *Fusobacteria*, and *Verrucomicrobia*. It is composed of six bacterial genera, accounting for more than 90% of the total intestinal flora, with genera of *Verrucomicrobia* accounting for 3–5% (31). Different dominant strains inhabit different intestinal sites, wherein interspecific competition and inhibition contribute to maintaining the equilibrium of the intestinal microenvironment.

Based on their different effects on the host, the bacterial taxa comprising the intestinal flora can be classified as pathogenic, beneficial, and neutral. The pathogenic bacteria, which can cause foodborne intestinal diseases, food poisoning, and other harmful diseases, include *Clostridium perfringens* and species of *Proteus* and *Salmonella*, which account for 10% of the total intestinal flora. Host-beneficial bacteria, accounting for approximately 20% of the total intestinal flora, include *Clostridium butyricum*, and species of *Akkermansia*, *Lactobacillus*, and *Bifidobacterium*, which play particularly important roles in the intestine that anti-inflammatory effect, mutual inhibition with harmful bacteria, etc. Neutral bacteria, also referred to as opportunistic pathogens, account for approximately 70% of the total number of intestinal bacteria, among which are *Escherichia coli*, *Lactobacillus*, *Streptococcus*, and *Veillonella*. Under normal conditions, these bacteria are generally harmless, although they can become pathogenic in response to the interference of adverse external factors (31–33). Increasingly, it is becoming evident that these gut bacteria play key roles in regulating multiple body processes, about which comparatively little is known at present, thereby highlighting the necessity of further in-depth research.

In recent years, studies have shown that the occurrence of diseases is closely related to the reduction of beneficial bacteria. Based on this research, people have made great achievements in the treatment of intestinal flora disorders by supplementing probiotics or supplementing synbiotics. Since grains are rich in components that promote the growth of beneficial bacteria (34, 35), we focused our attention on the beneficial effects of grains on intestinal flora.

### 1.2 Further research on intestinal flora can provide a reference for the occurrence of diseases related to intestinal flora

One of the primary contributions of intestinal flora is the production of beneficial metabolites, such as short-chain fatty acids (SCFAs), which mainly include acetic, propionic, and butyric acids. SCFAs can bind to the G-protein-coupled receptors (GPCR) GPR41 and GPR43, thereby inducing the secretion of glucagon-like peptide-1 (GLP-1), peptide YY (PYY), and leptin, which enhance the body’s energy metabolism (36). SCFAs can, to a certain extent, influence glucose and lipid metabolism by inhibiting the synthesis of fat, participating in immune regulatory processes, promoting intestinal digestion and absorption, reducing intestinal pH, and playing an important role in maintaining the balance of the intestinal flora (37). An unfavorable imbalance among intestinal flora can influence the nervous and immune systems via the brain–intestine–kidney and brain–intestine–bone marrow axes. Among the disorders that have been established to be associated with imbalanced intestinal flora is inflammatory bowel disease (IBD), in which patients are characterized by changes in the proportions, diversity, and composition of gut flora, which is reflected in an altered balance among pathogenic, conditional pathogenic, and beneficial bacteria, with increases in pathogenic types and reductions in beneficial bacteria (38). Generally, such imbalances between beneficial and pathogenic bacteria contribute to the development of diseases and disorders, and in this regard, some studies have found that changes in the intestinal microenvironment are reflected in increases or reductions in microbial metabolites, which in turn will influence the normal host metabolic activities and may be accompanied by metabolic disease (36). These findings accordingly highlight the close relationship between intestinal flora and human health, and thus, further research on intestinal flora could provide a reference for the occurrence of diseases associated with the activities of these microorganisms.

A number of cereal crop plants characterized by coarse grains have been established to influence the regulation of intestinal flora. For example, a study on polysaccharides derived from the coarse-grained Tartary buckwheat based *in vitro*-simulated fermentation found that these polysaccharides have a regulatory effect on intestinal flora and SCFA metabolites (39). Among these SCFAs, the contents of acetic, propionic, and butyric acids were observed to have increased during *in vitro* fermentation, which, compared with the control, was accompanied by corresponding increases in the abundance of probiotic flora, thus contributing to a regulation of the intestinal microenvironment. In a further study, Zhao et al. demonstrated that consumption of adzuki beans can alleviate obesity caused by a high-fat diet, which was found to be closely associated with the activities of intestinal flora (40). Compared with mice in a high-fat diet group, those consuming an adzuki bean-supplemented diet were found to have a significantly enriched bacterial flora in terms of abundance, although there were no obvious changes in bacterial diversity. The intestinal flora of mice fed the adzuki bean supplement was found to contain a greater abundance of probiotic strains, which partially compensated for the detrimental effects of a high-fat diet on the intestines of mice, and also contributed to a reduction in the biosynthesis of lipopolysaccharides. Accordingly, these findings indicate the positive effects of adzuki beans in regulating intestinal flora. Similarly, examination of the effects of different proportions of buckwheat in regulating the abundance of intestinal flora has indicated that high doses of dietary buckwheat can promote increases in the abundance of intestinal flora, reduce the intestinal absorption of cholesterol, and enhance the intestinal excretion of cholesterol (41). These findings accordingly provide important clues regarding the potential mechanisms whereby coarse-grain products can contribute to improving the intestinal microenvironment.

Based on the research conducted to date, it is speculated that the natural active constituents of quinoa can influence disease development by regulating intestinal flora. In this review, we accordingly focus on the regulatory effects of quinoa extracts and their active constituents on the composition and activity of intestinal flora.

## 2 The regulatory effects of quinoa bioactive components on intestinal flora

### 2.1 Saponins

The saponins in quinoa are characterized by their low bioavailability, poor absorption, and prolonged intestinal residence. These characteristics indicate the potential availability for metabolism by intestinal microorganisms and that they can serve as a source of secondary glycosides or aglycosides (42). Studies have shown that (43) Under the action of intestinal flora, quinoa saponin can be effectively hydrolyzed into sapogenin by the host, the release of sapogenin is related to the initial microbial composition of intestinal microflora, and the initial intestinal microflora interferes with the later conversion process of sapogenin of quinoa saponin through some mechanism. In addition, these saponins have been shown to play a beneficial role in regulating the intestinal flora in rats (44). A comparison of rats administered high, medium, and low doses of quinoa saponins revealed significant differences between the experimental and control groups with respect to the diversity of intestinal flora. The findings of metabolomic analyses revealed that these saponins influence multiple processes, including the metabolism of vitamin B_6_ and tryptophan and the ammonia cycle, thus regulating host metabolism and modifying the composition of the intestinal flora in rats. The findings of this study also provided evidence to indicate that in contrast to low doses, medium and high doses of quinoa saponins may have potentially toxic effects on the kidneys. These observations accordingly indicate that it would probably be safe to consume low amounts of quinoa saponins in the diet or quinoa-based food products.

Although an imbalance in the intestinal flora has been established to influence host metabolism (43), with metabolites having both beneficial and detrimental effects on the intestinal flora, the respective mechanisms of action remain unclear. Within the intestines, quinoa saponins can be effectively microbially hydrolyzed to yield sapogenin, the release of which has been shown to influence the initial microbial composition of intestinal microflora, which in turn interferes with the subsequent conversion of sapogenin. In this regard, Li et al. (42) have reported that total quinoa saponins have a bidirectional regulatory effect on intestinal microorganisms, with the ability to selectively promote the growth of beneficial bacteria. For example, treatment with quinoa saponins has been found to promote a significant increase in the relative abundance of *Lactobacillus*, has a beneficial effect on butyrate-producing bacteria, promotes health by enhancing the relative abundance of anti-inflammatory intestinal microbiota, inhibits the excessive growth of harmful bacteria, and reduces the production of lipopolysaccharides (LPS). Moreover, long-term supplementation with quinoa saponins is associated with anti-obesity effects by promoting a significant reduction in fat accumulation in obese rats, enhancing energy metabolism, and modifying the composition of intestinal flora. These findings also indicate a potential link between the composition of the intestinal flora and enhanced glucose tolerance and reductions in the levels of IL-6 and serum LPS levels. On the basis of these findings, it would thus appear that quinoa saponins have positive regulatory effects on intestinal flora.

Studies on rats, examining the effects of saponins derived from different varieties of quinoa have shown that quinoquinosides have membranolytic activity on small intestinal mucosal cells, with the effect being most pronounced with the quinoquinosides in bitter grains, and is associated with marked food aversion and impaired growth responses in rats. However, there is no clear evidence to indicate any deleterious effects on animal health (45). In a further study, Chen et al. extracted saponins from different colored varieties of quinoa and compared their compositions, digestion, and fermentation characteristics (46). They accordingly found that the quinoquinosides of black and white varieties had a higher saponin content than those from a red variety. The quinoquinosides of black quinoa were found to inhibit the growth of harmful bacteria (e.g., *Shigella* spp.) and promotes the growth of probiotic strains (*Lactobacillus* and *Bifidobacterium* spp.). Conversely, intestinal flora also releases sapogenin from quinoquinoside saponins. In a study assessing the safety of quinoa saponins and their effects on intestinal flora, in which rats were orally administered quinoa saponins for 90 days, the authors established that the maximum non-toxic dose of these saponins was in the range between 5 and 50 mg/kg (47). This result may be due to the consistent and inseparable relationship between the pathological characteristics of chronic kidney injury shown by the high-dose group of female rats after the experiment. More experiments are needed to verify the dose relationship between quinoa saponin and the body. Analysis of the intestinal flora of the treated rats revealed that the orally administered saponins promoted an increase in the number of intestinal flora species in female rats with relatively poor intestinal flora. Contrastingly, in male rats with relatively abundantintestinal flora, these saponins were found to alter the composition of the intestinal flora and also promoted an increase in the abundance of beneficial bacteria, whereas no corresponding increase was observed regarding the abundance of harmful bacteria. These findings thus provide further evidence to indicate that quinoa saponins have a positive effect with respect to improving intestinal flora, although the underlying mechanisms remain to be determined.

Ruoyu (44) and Zhang et al. (47) reported that quinoa saponin has no adverse effect on the host at a small dose of less than 5 mg/kg, on the contrary, at higher doses, such as 5 mg/kg-500 mg/kg, there is an adverse effect on the host. Under the influence of a higher dose of quinoa saponin, the host will cause kidney damage and chronic inflammation in the gastrointestinal tract. At present, more details of the safe dosage of quinoa saponin need to be further explored. In contrast, further exploration of the safe dosage range of quinoa saponin is of greater significance for the application of quinoa saponin. In addition to increasing the utilization of quinoa saponin, the related mechanism of saponin improving intestinal flora can also be further revealed.

### 2.2 Polyphenolic compounds

Among the polyphenolic constituents of quinoa, esculin has been identified as a polyphenol with medicinal properties. In a study using red junglefowl, Agarwal et al. examined the effects of quercetin 3-glucoside, 5% inulin, 5% quinoa cellulose, and other treatments on the microbiota populating the duodenum and cecum of these birds (48). The findings of duodenal 16rDNA analysis revealed that compared with the control group, treatment with 1% quinoa quercetin promoted increases in the numbers of in the group of probiotic microorganisms, reduced the numbers of potentially pathogenic *E. coli*, and increased the relative abundance of the dominant bacterial phylum *Firmicutes* in the intestinal flora. In addition, the combination of 1% quinoa quercetin and 5% quinoa cellulose resulted in an increase in goblet cells, which is good for the intestinal flora. The more goblet cells, the more mucin they produce, which strengthens the first line of defense of the intestinal flora (49). Another hypothesis is that acidic goblet cells may lower luminal pH, thereby influencing resident bacteria and directing the intestinal flora in a positive direction. These findings thus indicated that 1% quinoa quercetin has a prebiotic effect on the intestinal flora of red junglefowl, which could contribute to enhancing the composition of intestinal flora. Given the presumed pivotal role of intestinal flora in metabolic regulation, several studies have examined its effects on the metabolism of glucose and lipids, and in this regard, it has been established that the biological activity of quinoa polyphenols can also be manifested via their hypoglycemic effects mediated through inhibition of gastrointestinal digestive enzymes. In this process, quinoa polyphenols have been demonstrated to hinder the degradation of polysaccharides and retard the absorption of carbohydrates in the intestine by inhibiting α-amylase and α-glucosidase, which are key enzymes in the digestion and absorption of gastrointestinal sugars. Moreover, these compounds have also been found to contribute to lowering blood glucose levels (50). Collectively, these findings provide evidence indicating that quinoa polyphenols can enhance intestinal flora, which may be associated with the production of prebiotics and glucose metabolism-related mechanisms. Zhang et al. (51) reported that quinoa polyphenols could inhibit α-glucosidase activity in a dose-dependent manner through free polyphenols (FPE) and bound polyphenols (BPE). FPE and BRE inhibit α-glucosidase activity by binding to the enzyme site in a non-competitive mode and an un-competitive mod, thereby inhibiting the elevation of blood glucose level according to the previous reports on quinoa polyphenols, we speculate that the mechanism of quinoa polyphenols involved in intestinal flora may be that quinoa polyphenols inhibit enzymes involved in the regulation of the digestive tract, thereby affecting the abundance of intestinal flora and improving the microenvironment of intestinal flora. This also gives us an important hint that quinoa polyphenols have a great potential impact on the improvement of the body and provides strong support for the development and utilization of quinoa. The more detailed mechanism remains to be further explored.

### 2.3 Polysaccharides

The findings of previous studies have indicated that the extraction yield of quinoa polysaccharides differs among different cultivars, among which, the yield obtained from cultivar ‘NSL92331’ has been reported to be as high as high as 6.88% (52). Quinoa polysaccharides are considered prebiotics, and in this regard, simulated *in vitro* fermentation studies have revealed increases from 3.33 to 10.88% in the proportion of *Bifidobacteria* in a quinoa polysaccharide-treated group. Similarly, whereas *Actinobacteria* accounted for 5.28% of the intestinal flora in the control group, the proportion had increased to 16.98% in the quinoa polysaccharide group. Quinoa polysaccharides have been established to promote the growth of beneficial bacteria after fermentation, for example, *Bifidobacterium* and *Collinsella*, thereby indicating that these polysaccharides can be used as a crude source of fermented carbohydrates and have a significant prebiotic promotion effect on *Bifidobacterium* and other probiotic bacteria (53).

Quinoa polysaccharides can also contribute to ameliorating hyperlipidemia induced by consumption of a high-fat diet (HFD), the underlying process of which is regulated by intestinal flora. In a study in which Cao et al. examined the effects of quinoa polysaccharide supplementation in rats fed an HFD, the rats were orally administered a quinoa polysaccharide preparation (54). It was accordingly found that low-dose quinoa polysaccharide supplementation had regulatory effects on the bacterial community at the phylum, class, order, family, and genus levels. At the phylum level, the populations of *Firmicutes, Bacteroidetes*, and *Proteobacteria* were found to account for more than 90% of the total gut bacteria. However, this supplementation was observed to reduce the *Firmicutes* and increase those of *Bacteroidetes*, thereby leading to a reversal in the *Firmicutes* to *Bacteroidetes* ratio in the HFD group rats. Furthermore, in response to the low-dose polysaccharide treatment, there was an increase in the proportion of *Proteobacteria*, to levels similar to those observed in the normal control group. These findings thus provide further evidence supporting the role of quinoa polysaccharides as prebiotics in regulating intestinal flora. These reports indicate that quinoa polysaccharide has a positive effect on intestinal flora, and the related regulatory mechanism needs to be further explored. From the above reports, we also know that the imbalance of intestinal flora is not a simple process, but more a multi-sided regulation, and quinoa polysaccharide shows changes in intestinal flora, which also gives us favorable support for the development of quinoa.

Quinoa dietary fiber has been established to have a positive effect with respect to the regulation of intestinal flora, with SCFAs being notable beneficial products of the microbial fermentation of dietary fiber. The effects of quinoa bran soluble dietary fiber (QBSDF) on intestinal inflammation and homeostasis have been studied in mice with ulcerative colitis induced by sodium dextran sulfate (DSS) (55). Functional analyses of the intestinal flora in these mice revealed that QBSDF-induced improvements in the grouping, and structure of the intestinal flora community may include functional changes among these microbes. QBSDF was found to enhance the production of SCFAs (as determined from an analysis of fecal samples), attenuated DSS-induced dysbiosis of intestinal flora, and had positive effects on the maintenance of intestinal integrity. This fiber was also found to contribute to a significant enhancement of gut barrier function by promoting increases in the expression of tight junction proteins at both the mRNA and protein levels. Furthermore, the prebiotic properties of quinoa soluble cellulose have been confirmed based on an examination of the effects of intra-amniotic administration of 5% quinoa fiber to viable embryos mediated via its influence on the cecal bacterial population and intestinal morphology (45). Previous reports mentioned that there is another possible mechanism of quinoa polysaccharide related to SCFAs, which involves that quinoa polysaccharide may be related to the production of SCFAs during the fermentation process, which also involves the change of pH, thereby improving the living environment of beneficial bacteria and guiding the intestinal flora in a positive direction (Table 1).

**Table 1:** Characteristic differences among quinoa cultivars.

Characteristic	White	Red	Black	Ref.
Quinoa saponin antioxidant activity	+	++	+++	(11)
Saponins content	0.65 g/100 g	0.28 g/100 g	0.59 g/100 g	(46)
Sapogenin	24 g/100 g	4.66 g/100 g	24.82 g/100 g	
Polyphenolic substance				
The total concentration	466.99 ± 3.27 mg/kg	634.66 ± 5.87 mg/kg	682.05 ± 4.73 mg/kg	(56)
Minimum Inhibitory Concentration (MIC)	0.111–0.592 mg/mL	0.074–0.739 mg/mL	0.107–0.867 mg/mL	
Mechanisms of effects on intestinal flora	/	Inhibits the activity of α-glucosidase	/	(51)
Related reported diseases	/	Type 2 diabetic patient	/	
Inhibition of α-Glucosidase	+	++	+++	(57)
Inhibition of Lipase Activities	+	++	+++	
Quinoa polysaccharide				
Related reported diseases	HLP (hyperlipidaemia) and colonitis	/	/	(58)
Changes in the number of intestinal floras	Increase Bifidobacterium and Collinsella	/	/	

### 2.4 Bioactive peptides

Bioactive peptides are peptide compounds that have positive effects on biological activities, and whereas microorganisms can metabolize protein peptides, bioactive peptides have in turn, been established to have regulatory effects on the structure of intestinal flora (59).

Quinoa proteins are an important source of bioactive peptides. In an *in vivo* study of quinoa proteins in spontaneously hypertensive rats (SHR), analysis of the gastrointestinal contents revealed the presence of numerous promising bioactive peptide precursors released from quinoa proteins. Moreover, examination of fecal microbes in SHR treated with quinoa proteins revealed that these microbes have more common characteristics regarding genus composition than those in non-hypertensive rats (59). Quinoa proteins were found to promote a significant reduction in the blood pressure of SHR, an increase in bacterial alpha diversity (the number, evenness, and abundance of species), and a shift in the structure of intestinal flora to one closer to that of non-hypertensive rats. These findings accordingly indicate that quinoa proteins may serve as a potential therapeutic candidate for reducing blood pressure by ameliorating the intestinal flora dysbiosis associated with hypertension. In this regard, quinoa proteins have been identified as a promising natural source of angiotensin-converting enzyme (ACE) inhibitory peptides and can reduce blood pressure in SHR, in which the fecal microbiota undergo significant alterations. A further study on bioactive peptides derived from quinoa proteins has similarly confirmed that as precursors of bioactive peptides, quinoa proteins have regulatory effects on intestinal flora. The effects of quinoa proteins and the associated hydrolysate have also been investigated in an azoxymethane/dextran sulfate sodium (AOM/DSS)-induced mouse model of colorectal cancer (CRC) (60). Analysis of the intestinal flora and potential mechanisms associated with SCFAs showed that quinoa proteins or protein hydrolysates can alleviate the clinical symptoms of CRC and increase the contents of SCFAs in colonic tissues. In addition, these proteins, or the hydrolysate thereof, were found to partially alleviate an imbalance in the intestinal microbiota of CRC mice, which could be attributed to the fact that quinoa proteins can contribute to reducing the abundance of pathogenic bacteria and promote increases in the relative abundance of probiotic strains. Furthermore, PICRUSt analysis revealed that the functional characteristics of the intestinal flora in the quinoa protein-treated group were similar to those in the control group. Collectively, the findings of this study revealed that quinoa proteins and their hydrolysate can contribute to ameliorating AOM/DSD-induced CRC in mice, by altering intestinal flora and enhancing the production of beneficial SCFAs.

The findings of a further study conducted by Fotschki et al. have revealed that flour containing quinoa protein can significantly enhance cecal microbial activity, the activities of α-glucosidase, β-glucosidase, and α-galactosidase, and the production of SCFAs in rats, while promoting a reduction in the pH of digesters, thereby indicating the favorable effects of these proteins on growth parameters and metabolism of intestinal flora (61). Furthermore, in a study evaluating the effects of quinoa seeds supplemented with phytase and protease alone or in combination on microbial activity and intestinal flora, it was found that the addition of phytase and protease to the basal diet had beneficial effects on the growth performance, intestinal flora ecology, and gut morphology of broilers (62). The addition of protease supplements and phytase to a quinoa seed-based diet promoted increases in the viable cell counts of *Lactobacillus* and reduced those of *E. coli*, which could be associated with the effects of these enzymes on protein digestibility, thereby altering amino acid bioavailability and influencing microbial composition, as well the antimicrobial properties of protease supplements.

The diseases mentioned in these reports include spontaneous hypertension and colorectal cancer, both of which show a close relationship with intestinal flora in these reports. However, it is extremely strange that in a report by Zhang et al. (51), it is mentioned that α-glucosidase activity was inhibited. However, Fotschki et al. (61) report increased the activities of α-glucosidase, which is obviously in conflict. Nevertheless, since the diseases targeted by these two reports and the varieties of quinoa tested in these two reports are different, this may be explained. This also gives us some hints that, perhaps because of its various bioactive functions, quinoa is the good choice for a versatile grain in the face of so many different diseases (Table 2).

**Table 2:** The regulation mechanisms of quinoa bioactive peptides in different diseases.

Disease	Related mechanism	Ref.
Obesity	Regulation of the PPAR-α/γ signaling pathway in the liver and the community structure of intestinal flora	(65)
Spontaneously hypertensive	Angiotensin converting enzyme (ACE) inhibitory peptide is released	(59)
Colorectal Cancer (CRC)	Increased SCFAs content in colon tissue	(60)
Colorectal Cancer (CRC)	Inhibition of HDAC1 activity and regulation of cancer-related gene expression	(83)
Colonic inflammation	Quinoa protein and derived peptides inhibited TLR4 levels and IκB-α and NF-κB phosphorylation in colon tissues	(84)

**Figure 5:**
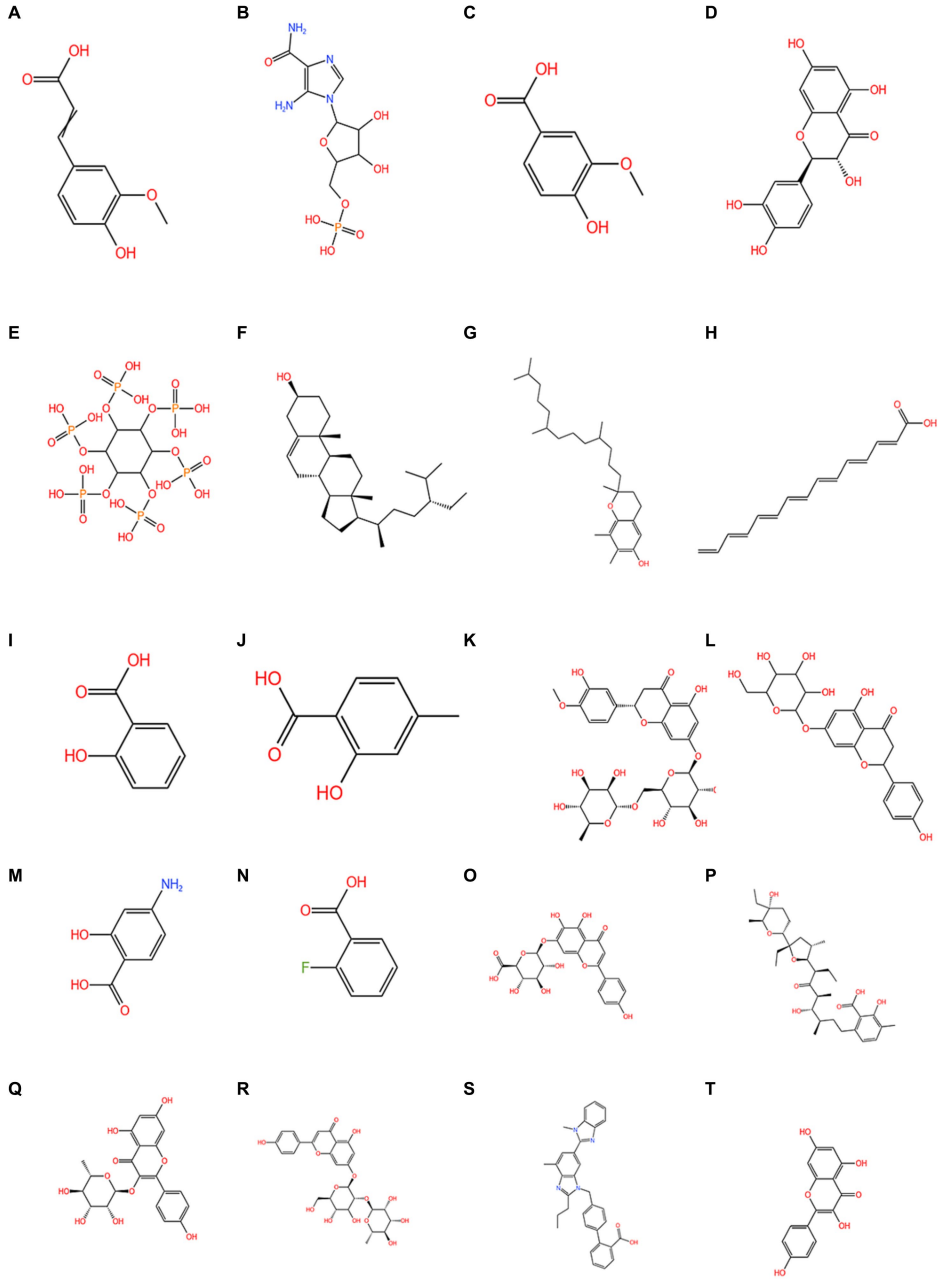
The chemical composition of quinoa. **(A)** 3-(4-hydroxy-3-methoxyphenyl)-2-propenoic acid; **(B)** 1h-Imidazole-4-carboxamide; **(C)** vanillic acid; **(D)** quercetin; **(E)** phytic acid; **(F)** phytosterol; **(G)** tocopherols; **(H)** C14-18 and C16-18-unsatd; **(I)** salicylic acid; **(J)** 4-methylsalicylic acid; **(K)** hesperetin 7-rhamnoglucoside; **(L)** naringenin-7-*O*-glucoside; **(M)** 4-aminosalicylic acid; **(N)** 2-fluorobenzoic acid; **(O)** scutellarin; **(P)** lasalocid; **(Q)** afzelin; **(R)** apigenin-7-*O*-neohesperidoside; **(S)** telmisartan; **(T)** kaempferol.

Figure 5 shows the molecular structures of the chemical components of quinoa (5, 6, 63, 64, 66–70).

### 2.5 Improvements in the intestinal flora promoted by whole quinoa extract

Studies that have evaluated the prebiotics in quinoa and amaranth have shown that these can promote the production of SCFAs, lactate, and formate during fermentation, which can serve as suitable substrates for intestinal flora metabolism (71). The high acetate concentrations obtained from the fermentation of both these substrates may be associated with increases in *Bifidobacterium*, *Proteus mirabilis*, or *Bacteroides*, thereby indicating potential prebiotic effects on the intestinal flora. Bianchi et al. have also demonstrated that a symbiotic beverage based on a soybean and quinoa water extract (30% quinoa and 70% soybean) had positive effects on the colon, promoting increases in the populations of beneficial *Lactobacillus* and *Bifidobacterium* and reducing those of potentially harmful genera, such as *Bacteroides* and *Clostridium* (72). Administration of this beverage also promoted a significant reduction in ammonia ion production and enhanced the diversity and richness of *Lactobacillus* spp. without influencing their activity. These findings thus indicate that quinoa and soy are good candidate plants for delivering probiotics to the intestinal flora, thereby enhancing the composition of the intestinal flora and hence improving human health (72). The findings of these and similar studies have thus confirmed that whole quinoa extracts can improve intestinal flora by yielding fermentation products beneficial to intestinal microbial metabolism, as well as through their roles as prebiotics. All the above reports show that quinoa has a certain role in promoting the health of intestinal flora. What can be observed now is that after the animal quinoa experiment, the beneficial bacteria increases, and the harmful bacteria decreases. All the reports seem to prove this. In addition, there is evidence in the signal transduction pathway, which also gives us a great hint. Perhaps from the molecular mechanism, we can further discover the improvement of quinoa on intestinal flora, and then expand the utilization value of quinoa.

### 2.6 The effects of quinoa on the intestinal flora in different hosts

In addition to studies on rodents and poultry, researchers have also assessed the intestinal flora-associated effects of quinoa in a range of other hosts. For example, Traughber et al. evaluated the effects of five ancient grains (including rice and quinoa) as carbohydrate sources on blood glucose and insulin levels in dogs (73). The intestinal flora of animals in the quinoa group were found to be characterized by an increase in the relative abundance of *Bacteroidetes*, and these authors established that diets comprising up to 40% of these grains, would have beneficial effects on overall host health, without adversely influencing nutrient digestibility, and had no significant effects on the levels of blood glucose or insulin in healthy dogs. These findings imply that quinoa-based diets might benefit dogs with obesity, insulin resistance, or diabetes. In a further study using alpacas, Carroll et al. examined the effects of four dietary supplements of quinoa, barley, amaranth, or soybean meal on the gastrointestinal tracts of these animals, and accordingly found that none of the four supplements significantly altered the composition of the compartment 1 C1 microbiota in alpacas. Moreover, they detected no significant difference between normal and poor alpacas with respect to the abundance of different (Operational Taxonomic Unit) orders (74). A study in humans consuming quinoa bread showed (75) that a small but not significant change in the type of grain consumed (whole grain quinoa instead of 20 g refined wheat flour) did not significantly modulate the intestinal flora, with a small but not significant increase in the relative abundance of Firmicutes in the control (quinoa free) and quinoa bread groups. Furthermore, in their study of the effects of quinoa extract on oviparous animals, Gee et al. examined the guts of chicks treated with quinoa extract (5% quinoa fiber) administered to the amniotic cavity of eggs containing viable embryos (45). Compared with the control group, this treatment was found to promote increases in the number of goblet cells in the villi and crypts. In addition, consumption of quercetin 3-glucoside and soluble quinoa fiber was found to have beneficial effects on the morphology of the duodenal brush border membrane and duodenal microbiota. In their study assessing the effects of quinoa supplementation in fish (76), Ahmed examined the histological structure of the intestinal wall in tilapia, and found that compared with the control group, fish fed a 20% quinoa-supplemented diet were characterized by significant increases in villus width and goblet cell number, although no significant differences were observed with respect to villus length. Thus, collectively, studies on the intestinal flora of a range of different hosts have provided evidence to indicate that consumption of quinoa can have positive regulatory effects on intestinal flora, which provides further potential clues for quinoa as a drug for intestinal flora regulation.

Table 3 summarizes recent research on quinoa in terms of disease treatment (58, 77–82).

**Table 3:** Research progress on quinoa in regulating intestinal flora of diseases.

Disease	Experimental procedure	Mechanisms	Changes in intestinal flora	Conclusion	Ref.
Obesity	Obese and normal mice were supplemented with whole quinoa, and the changes of intestinal flora were compared between the two groups.	1. Supplementation with the whole quinoa diet protected the body weight of mice. 3. Quinoa upregulated the expression of G protein bile acid-coupled receptor 5 (TGR5) and GLP-1 in the colon, brain, and downregulated the expression of TLR4 in the colon and liverandmarkers of ER stress and oxidative stress in the liver and serum. 4. The whole extract of Quinoa has a prebiotic effect, regulating intestinal flora. 5. The effect of quinoa whole extract on SCFAs production was mediated by intestinal flora.	1. Changes occurred in microbial abundance and metabolic profiles. 2. The number of Bacteroides, Actinomycetes and desulfurizing *Vibrio* were regulated. 3. The imbalance of intestinal flora was reversed.	1. The whole quinoa supplement diet may be associated with the delay of age-related metabolic diseases of obesity. 2. Quinoa can reduce the body weight, body fat, and fasting blood glucose of HFD obese mice, significantly improve obesity induced by high-fat diet, alleviate abnormal glucose and lipid metabolism, and reverse intestinal flora imbalance.	(76–78)
Liver cancer	Liver cancer was induced in mice and treated with protease inhibitor (pi)-rich salt solution obtained from quinoa.	1. Animals treated with pi showed higher hepatic MPO activity 2. Immune agonists promote hepatocarcinogenesis under pro-tumoral inflammation, reflecting to varying degrees an increase in the proportion of F4/80+ cells in the injured liver and a positive trend in the accumulation of immune mediators (CD68/CD206 ratio) in the intestinal tissue.	Appears to promote the positive impact of gut innate immune modulation on the functional outcome of the microbiome.	1. Potential effects of quinoa extract protease inhibitors on alleviating liver injury 2. Provides new insights into the immunomodulatory activity	(79)
Diabetes	Diabetic model mice were treated with a whole quinoa diet	1. Quinoa diet intervention reduced fasting blood glucose, body weight, blood lipid concentration, and insulin level; reduced HOMA-IR level; improved insulin tolerance and glucose tolerance; and significantly improved IR in db/db mice. 2. The effect of quinoa on T2DM is caused by the changes of intestinal flora and metabolism of the body.	The proportions of *Faecalibaculum* and *Lactobacillus reuteri* were increased.	1. Quinoa dietary intervention may ameliorate the diabetic process at the protein level. 2. Quinoa can effectively improve long-term hyperglycemia in diabetic mice, improve glucose tolerance, and enhance insulin sensitivity with few toxic side effects.	(80, 81)
Inflammatory bowel disease	Colitis was induced in a subset of mice from the groups fed AIN-93 M or whole quinoa diets.	1. Reduction in overgrowth of pathogenic bacteria. 2. Increase in gut species richness and diversity.	1. Reduced overgrowth of Escherichia/Shigella and *Clostridium difficile*. 2. Promote the growth of two genera of Lachnospiraceae.	1. Inhibit the imbalance of intestinal flora. 2. Quinoa has the potential to improve gut health. 3. Compared with DSS treatment, quinoa diet changed the intestinal flora more toward healthy intestinal flora.	(58, 82)

## 3 Summary and discussion

In this review, we discuss the potential applications of quinoa in enhancing the composition and activity of intestinal flora, with a specific focus on the different physiologically active constituents of this grain, namely, saponins, polyphenolic compounds, polysaccharides, bioactive peptides, and dietary fiber. Quinoa saponins can be hydrolyzed by the intestinal flora to yield sapogenin, which has been demonstrated to have positive effects regarding the abundance of probiotic bacteria. However, at high doses, the saponins in quinoa may have toxic side effects on the kidneys and may adversely influence the bacterial metabolism of vitamin B_6_ and tryptophan and the ammonia cycle of intestinal flora. Moreover, saponins derived from different varieties of quinoa characterized by different grain color have been found to have differing effects on intestinal flora. In animal experiments, quinoa quercetin was found to increase the number of probiotic bacteria (such as *Bifidobacterium* and *Phylum Firmicutes*) and reduce the number of pathogenic bacteria in the duodenum and cecum. This quercetin has also been established to have regulatory effects on the host through its hypoglycemic effects, mediated via the glucose and lipid metabolism of intestinal flora. As a crude source of carbohydrates, quinoa polysaccharides have been demonstrated to play an active role in ameliorating hyperlipidemia induced by high-fat diets, promoting the activity of probiotic strains, inducing changes in pH, and yielding SCFAs. In addition, quinoa proteins can be used as a source of bioactive peptide precursors to alleviate the symptoms of colorectal cancer in mice and enhance the production of SCFAs in colonic tissues. These proteins have also been found to promote the activities of microbial α-glucosidase, β-glucosidase, and α-galactosidase in the cecum, and contribute to reductions in cecal pH. Studies in oviparous animals have also shown that protein digestibility may alter amino acid bioavailability, thereby influencing intestinal flora composition.

Collectively, studies conducted to date on the bioactive constituents of quinoa have revealed their common effects in promoting the abundance of probiotic bacteria and inhibiting pathogens, as well as their contribution in reducing intestinal pH and promoting increases in the production of SCFAs and other key factors. Moreover, these effects have been demonstrated in a diverse range of hosts, including mammals, birds, and fish, and evaluations of the therapeutic potential of using whole-quinoa extracts have facilitated further progress in disease-related research. For example, with respect to obesity, inflammatory bowel disease, liver cancer, and diabetes, quinoa has been found to have beneficial effects on the body, mediated via its regulatory effects on the intestinal flora, which further highlights the central role of these microbes in host regulatory processes, including those associated with the gut–brain–liver and intestinal microbiota–brain routes. In addition, there is evidence to indicate that the constituents of different varieties of quinoa have slightly differing effects, which could be associated with the different growth environments of these varieties.

The research of quinoa in intestinal flora, on the one hand, the observation of intestinal flora in the biological activity function of quinoa, it is found that quinoa can promote the growth of beneficial bacteria and inhibit the growth of harmful bacteria, regulate some enzymes of intestinal flora, and then create an environment conducive to the growth of beneficial bacteria, reverse the environment of intestinal flora. For example, bioactive peptides or polyphenolic compounds of quinoa affect the expression of certain enzymes through signaling pathways, which in turn affect the intestinal flora or other diseases. The mechanisms by which quinoa improves the structure of intestinal flora need to be further explored, which may be multi-faceted, and the achievements in molecular mechanisms may give us some hints.

Based on the research conducted to date, quinoa has now reached a certain stage of therapeutic development. However, despite the significant progress in this regard, there is still a lack of relevant experimental data for the therapeutic efficacy of quinoa as a human drug. As indicated by the animal study findings, intestinal flora can be considered an important switch that quinoa can contribute to operating by regulating the constituent microbial populations. It is confidently predicted that with more in-depth research on quinoa, the utilization of this grain for medicinal purposes can be enhanced, and effective approaches will be developed for beneficially manipulating the intestinal flora. However, there are still many problems about the actual application of quinoa, such as the taste of quinoa and the control of quinoa saponin. We should not only tap the positive factors of quinoa, but also should not ignore the difficulties encountered in the reality of quinoa. Although the current market development of quinoa is not very good, with the in-depth research on the improvement mechanism of quinoa on intestinal flora, it is believed that the quinoa market will also have a new round of development in the near future, and what we need to do is to deeply explore the role of quinoa in the “homology of medicine and food”.

## Author contributions

HH: Investigation, Validation, Writing – original draft. CJ: Conceptualization, Investigation, Validation, Writing – original draft. XC: Formal analysis, Investigation, Writing – original draft. LZ: Investigation, Supervision, Writing – review & editing, Funding acquisition. YJ: Investigation, Supervision, Writing – review & editing. XM: Investigation, Supervision, Validation, Writing – review & editing. XL: Funding acquisition, Investigation, Project administration, Supervision, Validation, Writing – review & editing.
